# Human placental mesenchymal stem cells ameliorate liver fibrosis in mice by upregulation of Caveolin1 in hepatic stellate cells

**DOI:** 10.1186/s13287-021-02358-x

**Published:** 2021-05-20

**Authors:** Yunqi Yao, Zhemin Xia, Fuyi Cheng, Qingyuan Jang, Jiao He, Cheng Pan, Lin Zhang, Yixin Ye, Yuan Wang, Shuang Chen, Dongsheng Su, Xiaolan Su, Lin Cheng, Gang Shi, Lei Dai, Hongxin Deng

**Affiliations:** 1 State Key Laboratory of Biotherapy and Cancer Center/Collaborative Innovation Center of Biotherapy, West China Hospital, Sichuan University, Ke-yuan Road 4, No. 1, Gao-peng Street, Chengdu, 610041 Sichuan P.R. China; 2 Department of Obstetrics, Sichuan Provincial Hospital for Women and Children, Chengdu, P.R. China; 3 Department of Plastic and Burn Surgery, West China Hospital, Chengdu, P.R. China

**Keywords:** Caveolin-1, hPMSCs, HSCs, Liver fibrosis, TGF-/Smad pathway

## Abstract

**Background:**

Liver fibrosis (LF) is a common pathological process characterized by the activation of hepatic stellate cells (HSCs) and accumulation of extracellular matrix. Severe LF causes cirrhosis and even liver failure, a major cause of morbidity and mortality worldwide. Transplantation of human placental mesenchymal stem cells (hPMSCs) has been considered as an alternative therapy. However, the underlying mechanisms and the appropriate time window for hPMSC transplantation are not well understood.

**Methods:**

We established mouse models of CCl_4_-injured LF and administered hPMSCs at different stages of LF once a week for 2 weeks. The therapeutic effect of hPMSCs on LF was investigated, according to histopathological and blood biochemical analyses. In vitro, the effect of hPMSCs and the secretomes of hPMSCs on the inhibition of activated HSCs was assessed. RNA sequencing (RNA-seq) analysis, real-time PCR array, and western blot were performed to explore possible signaling pathways involved in treatment of LF with hPMSCs.

**Results:**

hPMSC treatment notably alleviates experimental hepatic fibrosis, restores liver function, and inhibits inflammation. Furthermore, the therapeutic effect of hPMSCs against mild-to-moderate LF was significantly greater than against severe LF. In vitro, we observed that the hPMSCs as well as the secretomes of hPMSCs were able to decrease the activation of HSCs. Mechanistic dissection studies showed that hPMSC treatment downregulated the expression of fibrosis-related genes, and this was accompanied by the upregulation of Caveolin-1 (Cav1) (*p* < 0.001). This suggested that the amelioration of LF occurred partly due to the restoration of Cav1 expression in activated HSCs. Upregulation of *Cav1* can inhibit the TGF-/Smad signaling pathway, mainly by reducing Smad2 phosphorylation, resulting in the inhibition of activated HSCs, whereas this effect could be abated if *Cav1* was silenced in advance by siRNAs.

**Conclusions:**

Our findings suggest that hPMSCs could provide multifaceted therapeutic benefits for the treatment of LF, and the TGF-/*Cav1* pathway might act as a therapeutic target for hPMSCs in the treatment of LF.

**Supplementary Information:**

The online version contains supplementary material available at 10.1186/s13287-021-02358-x.

## Background

Hepatic fibrosis is a reversible wound healing response caused by many chronic liver diseases, such as viral infection, alcohol abuse, and autoimmune hepatitis. It is characterized by activation of HSCs and excessive accumulation of extracellular matrix (ECM) in the liver [1, 2]. HSCs are a resident mesenchymal cell type located in the subendothelial space of Disse and usually display a quiescent state [3]. Following liver injury, HSCs are activated and transdifferentiated into the fibrogenic myofibroblasts, the major cell type that causes fibrosis and collagen synthesis. If the source of the injury is sustained, HSC activation and accumulation of ECM persist [4]. Advanced LF can develop into irreversible cirrhosis, portal hypertension, and liver failure, and correlates with an increased risk of hepatocellular carcinoma [5]. The available treatment methods mainly focus on inhibiting HSC activation and/or promoting ECM degradation. Although there are many drugs to treat LF, the effect is very limited and may also aggravate liver damage [1, 6]. Therefore, new strategies to delay or prevent the progression of LF are urgently required.

Cell therapy is a promising approach for the treatment of liver disease, hepatocyte-based therapies have been shown to be very effective in experimental animals, but limited cell sources and low proliferation have restricted their large-scale application [7, 8]. Recently, many studies have demonstrated the therapeutic potential of mesenchymal stem cells (MSCs) in liver disease [9, 10]. It has recently been shown that MSCs can secrete various cytokines in a paracrine manner to regulate inflammatory responses, hepatocyte apoptosis, and fibrosis, and finally restore liver function after acute injury or chronic fibrogenesis [11, 12]. Along with their properties of high self-renewal, multipotent differentiation capacity, and immunosuppressive qualities [13,15], MSCs are considered to be the most suitable source of cells for cell-based therapy for LF. Similarly, the secretomes of MSCs, which contain a large number of soluble proteins, free nucleic acids, lipids, and extracellular vesicles have been proved retain the same beneficial effect of the cell of origin for the treatment of LF [10, 16].

The placenta is another promising source of MSCs. In contrast to autologous MSCs, including bone marrow and adipose mesenchymal stem cells, human placental mesenchymal stem cells (hPMSCs) can be easily obtained in massive numbers by a simple and painless procedure [17, 18]. Furthermore, they exhibit greater self-renewal, multilineage differentiation capacity, and strong immunologic privileges [19, 20]. More importantly, many studies indicate that hPMSCs have therapeutic potential in liver diseases. A previous study proved the therapeutic potential of hPMSCs in miniature pigs model of acute liver failure [21]. Additional studies have shown that hPMSCs exert an anti-fibrotic effect when transplanted into rats with carbon tetrachloride (CCl_4_)-injured livers by promoting hepatic regeneration via increased autophagy [22]. Although the use of hPMSCs has been studied in animal models of LF, the number of MSCs transplanted and the appropriate time window as well as the mechanisms responsible for liver repair by hPMSCs are not well understood.

The aim of the present study was to investigate whether transplantation of hPMSCs reduces fibrosis in CCl_4_-injured mouse liver and to perform a comparative analysis of the important factors involved in MSC-based cell therapy. We further analyzed the involvement of the TGF-/*Cav1* pathway in hPMSC-mediated anti-fibrosis activity in vitro and in vivo. Our results provide further support that hPMSCs could provide a new avenue for the treatment of LF.

## Methods

### Isolation and identification of human placental-derived mesenchymal stem cells

Placental tissue was obtained from three health donors in the Sichuan Maternal and Child Health Hospital, upon consent of its donor according to procedures approved by the Medical Ethics Committee, Sichuan University (K2018109-1). hPMSCs were isolated and purified; the immunophenotype and differentiation potential of hPMSCs were then determined according to reported procedures [17, 20]. hPMSCs were cultured in mesenchymal stem cell basal medium (DAKEWE, Beijing, China) supplemented with 5% UltraGROTM (HPCFDCRL50, Helios), and cells between passage 3 and 6 were used for all experiments.

hPMSCs were examined for cell morphology, cell surface markers (CD105, CD73, CD90, CD166, CD11b, CD34, CD45, and HLA-DR), and performed pluripotency characterization for osteogenesis, lipogenesis, and chondrogenesis. The results were shown in Figure S1.

### HSC culture and in vitro study

Human primary hepatic stellate cells (HSCs) were provided by ScienCell Research Laboratories and cultured in Stellate Cell Medium (SteCM, San Diego, CA) supplemented with growth supplement (SteCGS). The TGF-1 (a well-known pro-fibrotic ligand) (20ng/mL)-mediated HSC activation were induced after growing in DMEM with only 0.2% FBS for 24 h and determined by western blot analysis (Figure S3). To investigate the effect of hPMSCs in vitro, hPMSC secretomes were harvested and concentrated 15-fold with ultrafiltration tubes (millipore), activated HSCs were cultured with concentrated hPMSC secretomes in a gradient ratio (10%, 20%, 40%). Additionally, to exclude the effect of medium compositions, concentrated MSC complete medium also treated in the same manner, and the results were compared to the hPMSCs supernatant. Unactivated and activated HSCs without extra treatment were used as controls.

### CCl_4_-injured mice liver fibrosis and hPMSC transplantation

To induce liver fibrosis, 8-week-old C57BL/6 mice (20 2 g) were intraperitoneally injected with CCl_4_ (0.5 mL/kg body weight, dissolved in olive oil, 1:9; Sigma-Aldrich) twice a week for 6 weeks (*n* = 20). Five mice were sacrificed every 2 weeks for histopathological examination and liver function test. The liver tissue sections from CCl_4_-treated mice exhibited focal fibrosis, confirming the successful establishment of an animal model of liver fibrosis. No animals died during the experiments after CCl_4_ administration. All experimental procedures involving animal experiments were approved by the Sichuan University Medical Ethics Committee (K2018109-2).

**Fig. 1:**
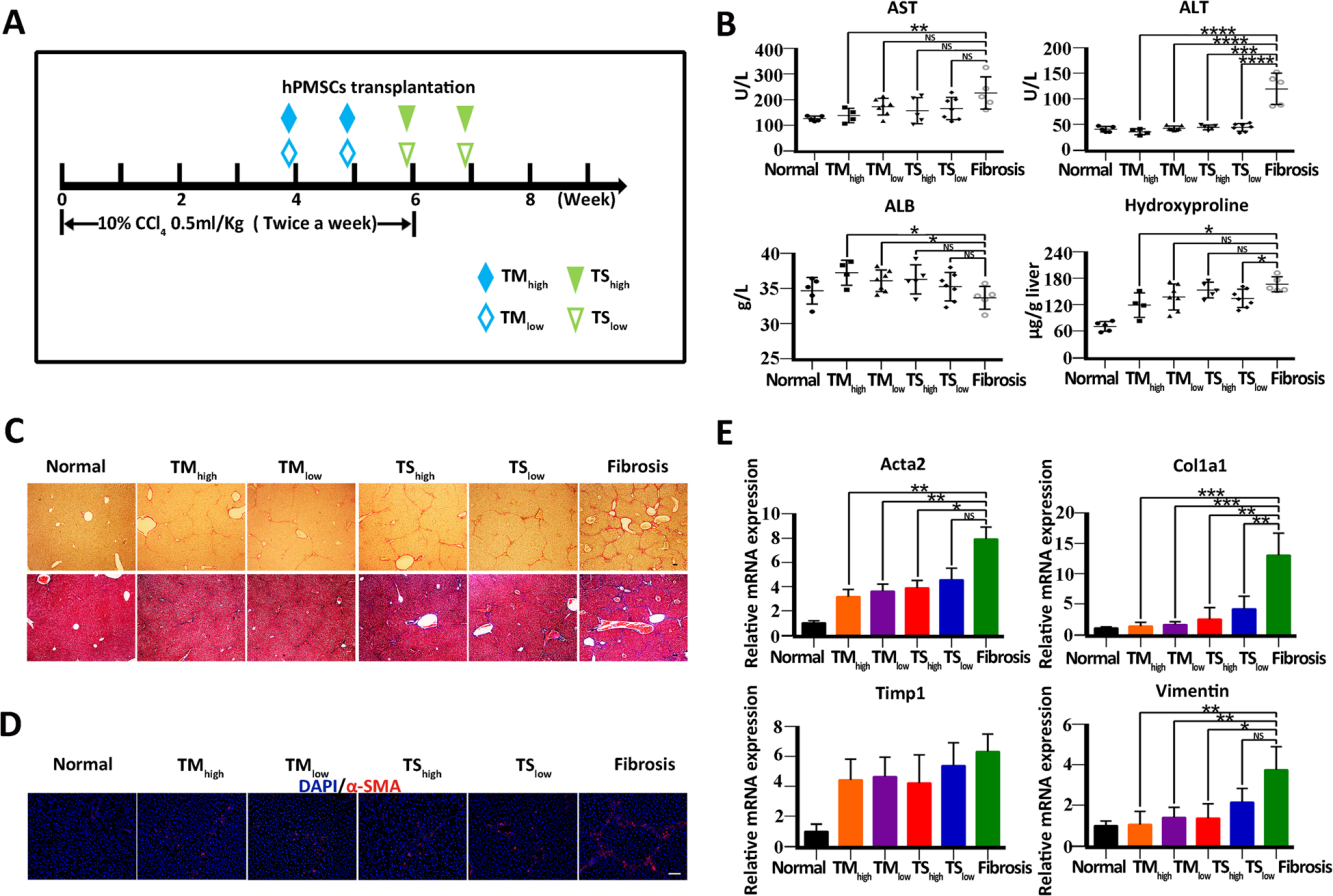
Therapeutic effects of hPMSCs in CCl_4_-induced liver fibrosis. **a** Experimental scheme of hPMSC transplantation in CCl_4_-injured liver fibrosis. Intravenous injection of hPMSCs was administered. TM_high_, treatment with a high dose of hPMSCs in the mild-to-moderate stage of LF; TM_low_, treatment with a low dose of hPMSCs in the mild-to-moderate stage of LF; TS_high_, treatment with a high dose of hPMSCs in the severe stage of LF; TS_low_, treatment with a low dose of hPMSCs in the severe stage of LF. A high dose was specified as 5 10^7^ cells/kg and a low dose was specified as 2 10^7^ cells/kg. **b** Hepatic function was assessed by serum level of AST, ALT, ALB, and hepatic hydroxyproline content in liver tissues in CCl_4_-injured mice that treated with or without hPMSCs. **c** Photomicrographs of liver sections stained with Sirius red (upper) and Masson trichome (bottom) at week 8. **d** Immunohistochemical staining using anti--SMA (red) and DAPI (blue) at week 8. **e** Expression of *Acta2, Col1a1, Timp1*, and *Vimentin* was determined using qRT-PCR. Relative mRNA expression was normalized to *-actin* and compared with the fibrosis group. Mice from fibrosis group received PBS followed by CCl_4_ injection. Scale bar, 50 m. ****p < 0.0001, ***p < 0.001, **p < 0.01, *p < 0.05; ns, no significance, hPMSCs, human placental mesenchymal stem cells. TM_high_ mice, *n* = 4/group; normal, TS_high_, and Fibrosis mice, *n* = 5/group; TM_low_ and TS_low_ mice, *n* = 7/group

In this study, hPMSCs were injected into the tail vein of mice in the liver fibrosis model after 4 or 6 weeks of CCl4 treatment, once a week for 2 weeks. These two time points of treatment with hPMSCs: mild-to-moderate stage of LF and severe stage of LF, corresponding to TM and TS, respectively. Meanwhile, the different doses of hPMSCs (high dose, 5 10^7^ cells/kg; low dose, 2 10^7^ cells/kg) were also tested in each group, as shown in Fig. 1a. The liver fibrosis group mice were injected with PBS alone. Untreated mice were treated as control. There were four hPMSC treatment groups in Fig. 1 as TM_high_, TM_low_, TS_high_, and TS_low_. The default for all hPMSC groups in the subsequent in vivo experiments was TM_low_.

**Fig. 2:**
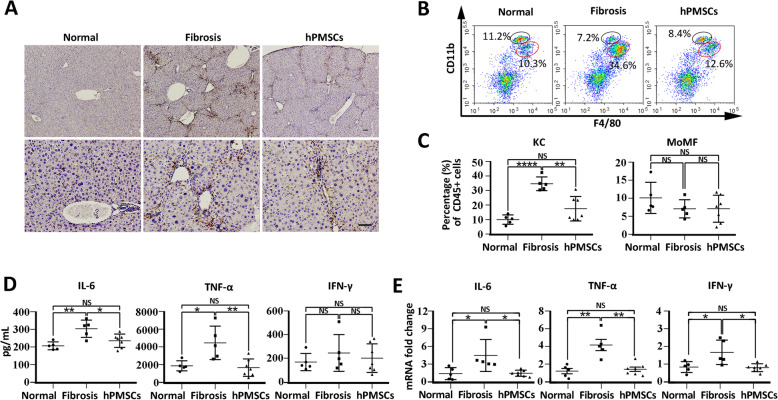
hPMSCs improve inflammation microenvironment in CCl_4_-induced liver fibrosis. **a** Immunohistochemistry staining using anti F4/80 antibodies at 2 weeks after the second injection of hPMSCs. **b** Monocyte-derived macrophage (MoMF) or Kupffer cells (KC) isolated from liver tissues were detected by FCAS analysis. MoMFs (CD11b^high^F4/80^low^) were inside the black circle and KCs (CD11b^low^F4/80^high^) were inside the red circle. **c** A statistical analysis was performed to determine the proportion of MoMF or KC in CD45+ cells. **d** Inflammatory cytokines in serum (IL-6, TNF-, IFN-) were examined with ELISA assays. **e** Expression of inflammatory cytokines mRNA was determined using qRT-PCR. Relative mRNA expression was normalized to *-actin* and compared with the fibrosis group. Mice from fibrosis group received CCl_4_ followed by PBS injection. Scale bar, 50 m. ****p < 0.0001, ***p < 0.001, **p < 0.01, *p < 0.05; ns, no significance. Normal and fibrosis mice, *n* = 5/group; hPMSCs mice, *n* = 7/group

Serum and liver tissue samples were collected at 2 weeks after the 6-week course of CCl_4_ administration. Specifically, samples were collected at the same time for mice of all groups, including normal, fibrosis, TM_high_, TM_low_, TS_high_, and TS_low_ group in Fig. 1 and normal, fibrosis, and hPMSC groups in Fig. 2 and in Fig. 6.

### Western blot analysis

Total proteins were extracted from HSCs with different treatments, equal amounts of soluble protein were separated via sodium dodecyl sulphate-polyacrylamide gel electrophoresis using 10% Tris-glycine mini-gels and transferred onto a nitrocellulose membrane (Bio-Rad).The primary antibodies were listed in Table S1, GAPDH mAb (Santa Cruz, Biotechnology) was used as an internal control. Following incubation with horseradish peroxidase-conjugated secondary antibody (Zsbio, Beijing, China) for 2 h at room temperature, the bands were then tested by a chemiluminescent substrate ECL kit (Merck Millipore).

### Flow cytometry and immunofluorescence staining

Immunophenotype of hPMSCs and intrahepatic macrophages were detected by flow cytometry. The single-cell suspensions were filtered, fixable Viability Stain 620 (BD Biosciences) were used to discriminate live and dead cells. The cells were then blocked with Fc-block (BD Biosciences) and stained with fluorochrome-labeled mAbs. Data were acquired on a NovoCyte flow cytometer. The primary antibodies were listed in Table S1.

For immunofluorescence staining, the cells were fixed in 4% paraformaldehyde for 20 min. The fixed cells were blocked with goat serum and subsequently incubated with primary antibodies at 4 C overnight. After thorough washing, secondary antibodies were used. Nuclei were visualized with DAPI (Roche Basel, Switzerland). The primary antibodies were listed in Table S1.

### Quantitative real-time reverse transcription-polymerase chain reaction (qRT-PCR)

Total RNA was isolated from liver tissue and other cells using Trizol reagent (Invitrogen, USA). qRT-PCR was performed using Step-One Real-Time PCR system (Takara) according to the manufacturers instructions. The expression of genes was normalized to GAPDH. All reactions were repeated in triplicate, and the primer sequences were listed in Tables S2 and S3.

### Microarray analysis

Total RNA of HSCs or liver tissues obtained from different groups was prepared with Trizol reagent (Invitrogen, Carlsbad, CA, USA). The products were sequenced by an Illumina HiSeq2500 instrument in Shanghai Majorbio Biopharm Technology Co. Ltd. (Shanghai, China). Data were extracted and normalized according to the manufacturers standard protocol [23, 24]. Differentially expressed genes were identified using the nbinomTest and DESeq (2012) functions estimate Size Factors. Genes displaying twofold or greater changes (*P* < 0.05, *t* test) in expression level between control group and test group were selected to generate the heatmap and for GO term enrichment analysis.

The RNAseq raw expression files and details of liver tissues have been deposited in NCBI GEO under accession nos. SRR12777460, SRR12777461, and SRR12777462. The RNAseq raw expression files and details of HSCs have been deposited in NCBI GEO under accession nos. SRR12806194, SRR12806195, SRR12806196, and SRR12806197.

### Liver function tests and histological analysis

Liver function and fibrotic degree were assessed by analyzing the levels of serum aspartate aminotransferase (AST), alanine aminotransferase (ALT), albumin (ALB), and hydroxyproline using UniCel DxC 800 Synchron (Beckman Coulter) according to the manufacturers instructions.

For histopathological and immunohistochemical analysis, formalin-fixed, paraffin-embedded liver samples were cut into 4-m-thick sections and stained with hematoxylin-eosin (H&E), Sirius red, and Masson staining. At least 3 animals per group were examined. The primary antibodies were listed in Table S1.

### Enzyme-linked immunosorbent assay

The serum of the mice in each group was separated and detected according to the manufacturers instructions of Xinboshengs QuantiCyto Mouse ELISA kits. IL-6, TNF-, and IFN- were tested.

### Transfection of *Cav1* small interfering RNA in HSCs

To investigate the role of *Cav1* in inhibiting the activation of HSCs, we designed three small interfering RNA (siRNAs) and transfected into HSCs with the Invitrogens Lipofectamine 3000 reagent. The downregulation of *Cav1* in HSCs was determined by western blot analysis and qRT-PCR. siRNA-negative control (siRNA-NC) and untransfected HSCs were treated as controls. The sequences of siRNAs were listed in Table S4.

### Statistical analyses

For statistical analysis, the experimental data were analyzed by SPSS software version 17.0 statistical software. Multivariate data were compared using analysis of variance. After statistical significance, pairwise comparisons were performed by Sidaks multiple comparisons test. All statistical graphs were drawn using Prism 6.0 (GraphPad). *p* 0.05 was considered significant.

## Results

### hPMSC transplantation alleviated CCl_4_-injured liver fibrosis in mice

In order to evaluate the effect of hPMSCs in the treatment of LF, we tested the efficiency of hPMSC engraftment in experimental LF by CCl_4_ administration, as shown in the modeling process (Figure S2 A). After CCl_4_ treatment for 2 weeks, normal liver lobules were destroyed and the fibrous connective tissue in the portal area was significantly increased (Figure S2 B-C). Four weeks later, the fibrous tissue further increased, extending to adjacent liver lobules and dividing the liver tissue. Six weeks later, the increased collagen fibers formed linear fibrous septa, and pseudolobules formed, according to Sirius Red staining, Masson staining, and -SMA staining (Figure S2 B-C). AST, ALT, and hepatic hydroxyproline content were also elevated in CCl_4_-treated mice, while the ALB levels decreased, with the trend consistent with histopathological examinations (Figure S2 D).

Time-point and cell-dose are two important parameters for cell-based therapy. In previous studies, MSCs were usually transplanted in vivo at the 4^th^ week or 6^th^ week after CCl_4_ administration [25, 26]. They reflect the mild-to-moderate stage of LF and the severe stage of LF, respectively. In this study, these two time points (TM and TS) were chosen respectively, and a comparative study was conducted, as shown in Fig. 1a. Two doses of hPMSCs, including high cell doses (5 10^7^ cells/kg) and low cell doses (2 10^7^ cells/kg) were analyzed as well, mice from fibrosis group received PBS followed by CCl_4_ injection were served as control. Compared with the fibrosis group, biochemical parameters of liver function, including ALT and AST levels were reduced, while the ALB levels increased in all hPMSC treatment groups, especially in the TM groups (Fig. 1b). Moreover, we found that the level of hydroxyproline, the main component in collagen tissue, was also reduced in all hPMSC treatment groups (Fig. 1b).

Histopathological examination using Sirius Red staining and Masson staining was performed to quantify the degree of LF. Compared to the fibrosis group, the fibrous area of liver tissue was significantly reduced in the hPMSC treatment groups (Fig. 1c). In addition, immunostaining showed -SMA expression was decreased in hPMSC treatment groups (Fig. 1d). Furthermore, the expression of fibrosis-related genes, including *Acta2*, *Col1a1,* and *Vimentin* was decreased upon hPMSC treatment, and downregulation of these genes was greater in TM groups compared with TS groups, as determined by qRT-PCR analysis (Fig. 1e). These results suggest that hPMSC treatment could improve liver function and alleviate LF in CCl_4_-treated mice.

Compared with the TS groups, the therapeutic effects of hPMSC transplantation were more profound in TM groups according to the results of the blood biochemical indices, collagen area, and fibrosis-related genes. Moreover, increasing the cell doses from TM_low_ to TM_high_, did not further improve the therapeutic effect of hPMSCs, indicating that low cell doses (2 10^7^ cells/kg) are sufficient to play a therapeutic role in experimental LF (Fig. 1be). The default for all hPMSC groups in the subsequent in vivo experiments was TM_low_.

### hPMSC transplantation has an anti-inflammatory effect in CCl_4_-injured mice liver

Inflammation is vital to the initiation and progression of LF [1]. To investigate the potential anti-inflammatory effects of hPMSCs in vivo, we examined the differences in intrahepatic macrophages and inflammatory cytokines in liver tissue isolated from different mice. Compared to normal liver tissue, a large number of infiltrating macrophages (F4/80^+^) were found in fibrotic livers according to immunohistochemical staining, while the number of macrophages reduced with hPMSC treatment (Fig. 2a). To explore the source of infiltrating macrophages, we then examined the proportion of mononuclear/macrophage cells using FACS analysis. The results showed that there was no statistical difference in the proportion of monocyte-derived macrophages (MoMF, CD11b^high^F4/80^low^) [27] from different liver tissues. Interestingly, the proportion of Kupffer cells (CD11b^low^F4/80^high^) [27] was 17.44 3.18% in the hPMSC group, which was significantly lower than that in the fibrosis group (34.64 2.12%) (Fig. 2b, c). These results suggested that hPMSC treatment could suppress the infiltration of macrophages, mainly by reducing the number of Kupffer cells.

We then detected the expression of inflammatory cytokines in the serum of mice by ELISA. As expected, the expression levels of inflammatory factors, including IL-6 and TNF-, were lower in the hPMSC treatment group than in the LF group (Fig. 2d). However, there was no significant change (*P* = 0.07) in the expression level of IFN-, which may be related to the alleviation of inflammation in mice after treatment. These results were further confirmed by qRT-PCR analysis (Fig. 2e). Our findings indicated that hPMSC treatment contributes to the improvement of CCl_4_-injured LF, at least in part through anti-inflammatory processes.

### hPMSCs inhibit TGF-1-induced HSC activation in vitro

**Fig. 3:**
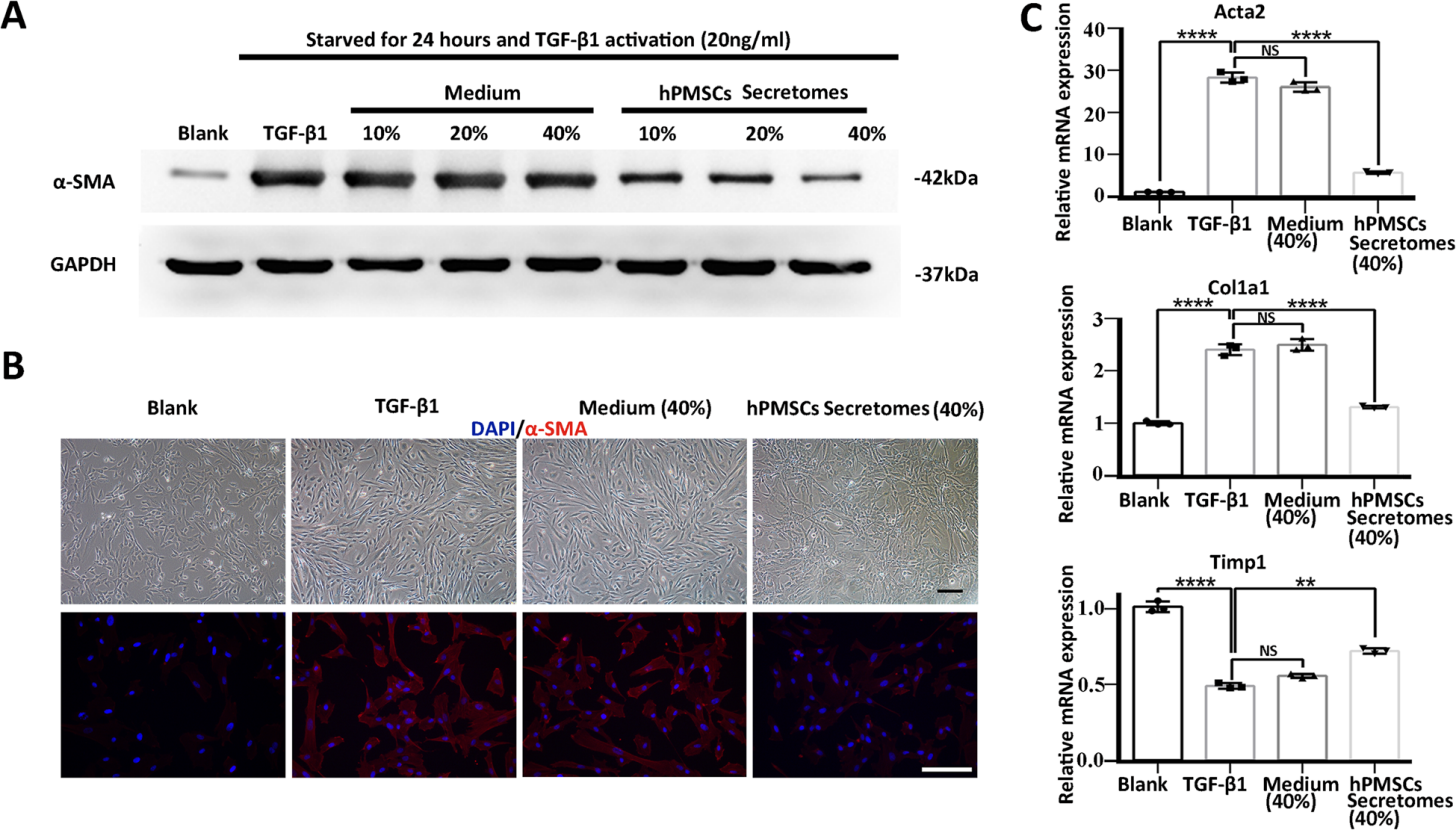
The activation of HSCs was inhibited by hPMSC in vitro. **a** Representative western blot of -SMA and GAPDH of activated HSCs, in the presence of concentrated MSC medium or concentrated hPMSC culture supernatant (hPMSC secretomes). **b** Typical cell morphology (upper) and -SMA immunofluorescence staining (lower) of HSCs. **c** Expression of fibrosis-related genes in activated HSCs was determined using qRT-PCR. Relative mRNA expression was normalized to *-actin* and compared with the TGF-1 group. Cells from blank group were unactivated HSCs, cells from TGF-1 group were activated HSCs that induced by TGF-1, and cells from medium group or hPMSC group were activated HSCs treated with either concentrated MSC medium or hPMSC secretomes. Scale bar, 50 m. ****p < 0.0001, ***p < 0.001, **p < 0.01; *p < 0.05; ns, no significance. HSCs, hepatic stellate cells

HSC activation is an indispensable component in the initiation and progression of LF [3]. TGF-1 treatment is a common way to activate resting HSCs in vitro [4]. In the presence of TGF-1, the number of activated HSCs that expressed the myogenic marker -SMA was markedly increased, as determined by western blot analyses (Fig. 3a; Figures S3; S4 A). Activated HSCs became elongated, with a dendritic-like shape, compared with unactivated HSCs (Fig. 3b; Figure S4 B).

Recent studies now support that the beneficial effects observed with MSC-based therapy can be mediated through the paracrine release of soluble proteins or other biologically active molecules and extracellular vesicles, which together constitute the MSC secretomes [6, 16, 25, 28, 29]. Therefore, we investigated whether hPMSCs and their secretomes could regulate the activity of the HSCs in vitro. In the present study, hPMSCs were co-cultured with activated HSCs. Meanwhile, the secretomes, which are 15-fold concentrations of the culture supernatant from hPMSCs, were cultured with activated HSCs in a gradient ratio of 10%, 20%, and 40%. Unactivated and activated HSCs without extra treatment were used as controls. Western blot (Fig. 3a; Figure S4 A) and immunofluorescence staining showed that -SMA levels were significantly decreased, and this was accompanied by changes in cell morphology that resulted in a morphology similar close to that of unactivated HSCs (Fig. 3b; Figure S4 B). The suppression of HSC activation was also confirmed by qRT-PCR analysis, which revealed a restoration of fibrosis-related genes in the hPMSCs and hPMSC secretomes group compared with activated HSCs. In particular, *Acta2*, the -SMA coding gene, was significantly downregulated compared with the activated HSCs, while the expression of *Timp1*, an anti-fibrotic gene was increased with hPMSCs and hPMSC secretomes treatment (Fig. 3c; Figure S4 C). These results demonstrate that HSC activation can be inhibited by treatment with hPMSCs and hPMSC secretomes.

Interestingly, the expression level of -SMA protein in activated HSCs was downregulated after treatment with 10% hPMSC secretomes. This was the same effect that was seen with hPMSC co-culture treatment. Furthermore, this inhibitory effect was more pronounced as the secretome concentration was increased to 40%, indicating that hPMSC secretomes inhibited HSC activation in a concentration-dependent manner (Fig. 3a, b). Additionally, to exclude the effect of MSC medium composition, MSC complete medium was also concentrated and tested in the same manner. Compared with the hPMSC supernatant, the concentrated MSC medium (40%) exhibited limited effects on the inhibition of HSC activation (Fig. 3a, b). The default for all hPMSC groups in the subsequent in vitro experiments was 40% hPMSC secretome concentration.

### *Cav1* is a potential target for hPMSC treatment in liver fibrosis

**Fig. 4:**
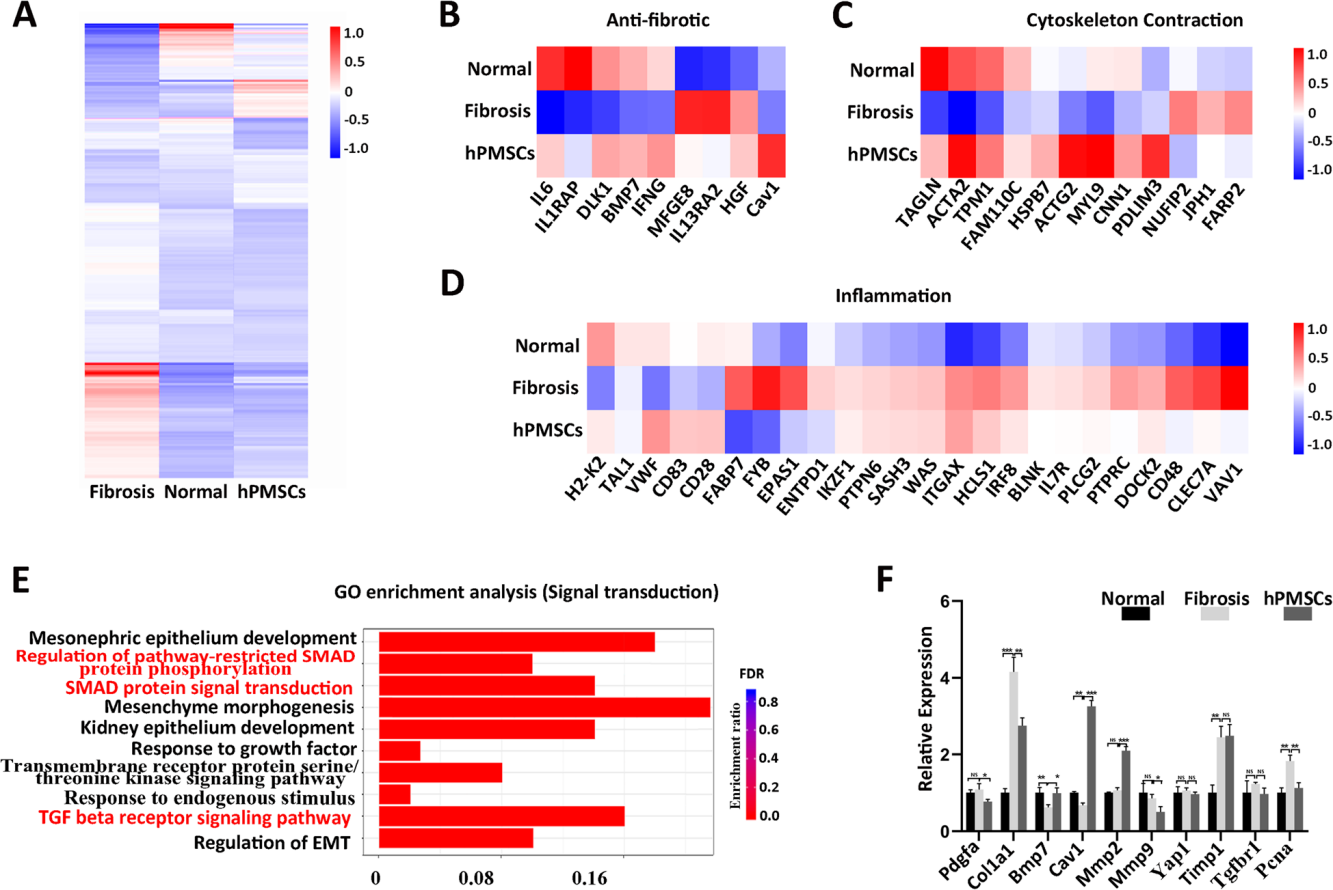
Cav1 participates in the improvement of CCl_4_-injured liver fibrosis after hPMSCs treatment. **a** Heatmap showing the differentially expressed genes (DEGs) among liver tissues from hPMSC group, normal, and fibrosis group (log2 fold change 2, *p* < 0.05). **bd** Heatmaps of three key gene clusters associated with liver fibrosis. **e** GO analysis (biological processes) of significantly downregulated DEGs between hPMSC group and fibrosis group. (*p* < 0.05). **f** Expression of ten fibrosis-related genes at different groups was determined using qRT-PCR. Relative mRNA expression was normalized to *-actin* and compared with the fibrosis group. Mice from fibrosis group received PBS followed by CCl_4_ injection. ***p < 0.001, **p < 0.01, *p < 0.05; ns, no significance; *Cav1, Caveolin-1*

To further investigate the mechanism of relieving LF with hPMSC treatment, we performed RNA sequencing (RNA-seq) analysis of liver tissues obtained from normal C57 mice (Normal group), mouse models with hepatic fibrosis (Fibrosis group), and hPMSC-treated fibrosis mice (hPMSC group). RNA-Seq analyses showed that the gene expression profiles of the hPMSC group was more closely resembled those seen in normal liver tissues and were significantly different from those fibrosis group (Fig. 4a). Furthermore, the genes included in three key functional clusters, including fibrosis, cytoskeleton, and inflammation-related factors were analyzed. The results revealed a significant change in the expression of these genes in fibrotic liver tissues, and they can be restored after hPMSC treatment (Fig. 4bd).

In addition, the top 10 GO biological process terms are listed in Fig. 4e. The terms related to TGF- signaling pathway, SMAD protein signal transduction and SMAD protein phosphorylation in this list suggests that regulation of TGF-/Smad signaling may be a potential mechanism in the treatment of LF with hPMSCs. We also performed qRT-PCR analyses to check the expression of ten important fibrosis-related genes (Fig. 4f). Among them, *Cav1* revealed the most significant differences between fibrosis group and hPMSC group. Importantly, previous studies have shown that *Cav1* can participate in regulating TGF-/Smad signaling pathway in many situations [30], indicating that *Cav1* may be a potential target for hPMSC treatment in LF.

**Fig. 5:**
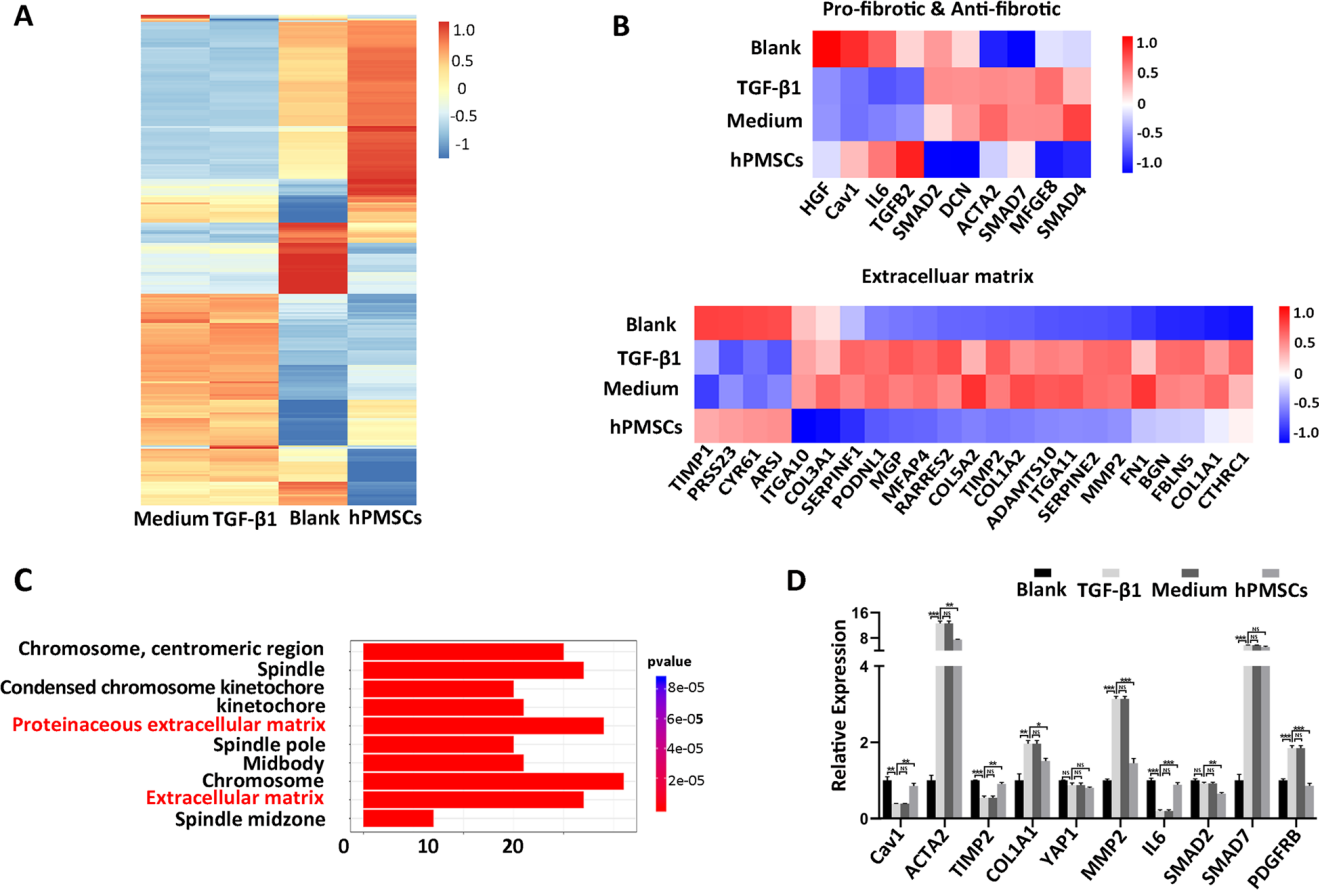
Cav1 is a potential target of hPMSC secretomes in inhibition of activated HSCs. **a** Heatmap showing the DEGs among HSCs from hPMSC group, blank group and medium group (log2 fold change 2, *p* < 0.05). **b** Heatmaps of two key gene clusters associated with HSC activation and liver fibrosis. **c** GO analysis (biological processes) of significantly downregulated DEGs between hPMSC group and TGF-1 group (*p* < 0.05). **d** Expression of ten fibrosis-related genes at different groups was determined using qRT-PCR. Relative mRNA expression was normalized to *-actin* and compared with the fibrosis group. Cells from blank group were unactivated HSCs, cells from TGF- group were activated HSCs that were pretreated with TGF-, and medium group or hPMSC group were activated HSCs treated with either MSC medium or hPMSC secretomes. ***p < 0.001; **p < 0.01; *p < 0.05; ns, no significance

To further confirm the above findings, we performed RNA-seq analyses on different cells, including activated HSCs (TGF-1 group), unactivated HSCs (Blank group), and activated HSCs, which were then treated with hPMSC secretomes (hPMSC group) and medium (Medium group), respectively. The gene expression profiles of the hPMSC group more closely resembled those seen in blank group and were significantly different from those TGF-1 group and medium group (Fig. 5a). Furthermore, the expression of fibrosis-related genes as well as the ECM-associated genes was similar to findings in liver tissue. It appears that, hPMSC secretomes can restore the expression of genes that had changed in the TGF-1 group or medium group to some extent (Fig. 5b). In addition, the top GO biological process terms related to the proteinaceous ECM and ECM suggest that hPMSC secretomes may reduce the formation of ECM by inhibiting the activation of HSCs, a key factor in alleviating LF (Fig. 5c). We also performed qRT-PCR analyses to check the expression of ten important fibrosis-related genes (Fig. 5d). The expression of *Cav1* was significantly upregulated in activated HSCs when cultured with hPMSC secretomes, further supporting the finding of vivo analysis, indicating that *Cav1* might be a potential target for hPMSC treatment in LF.

### Downregulation of *Cav1* is associated with activation of HSCs

**Fig. 6:**
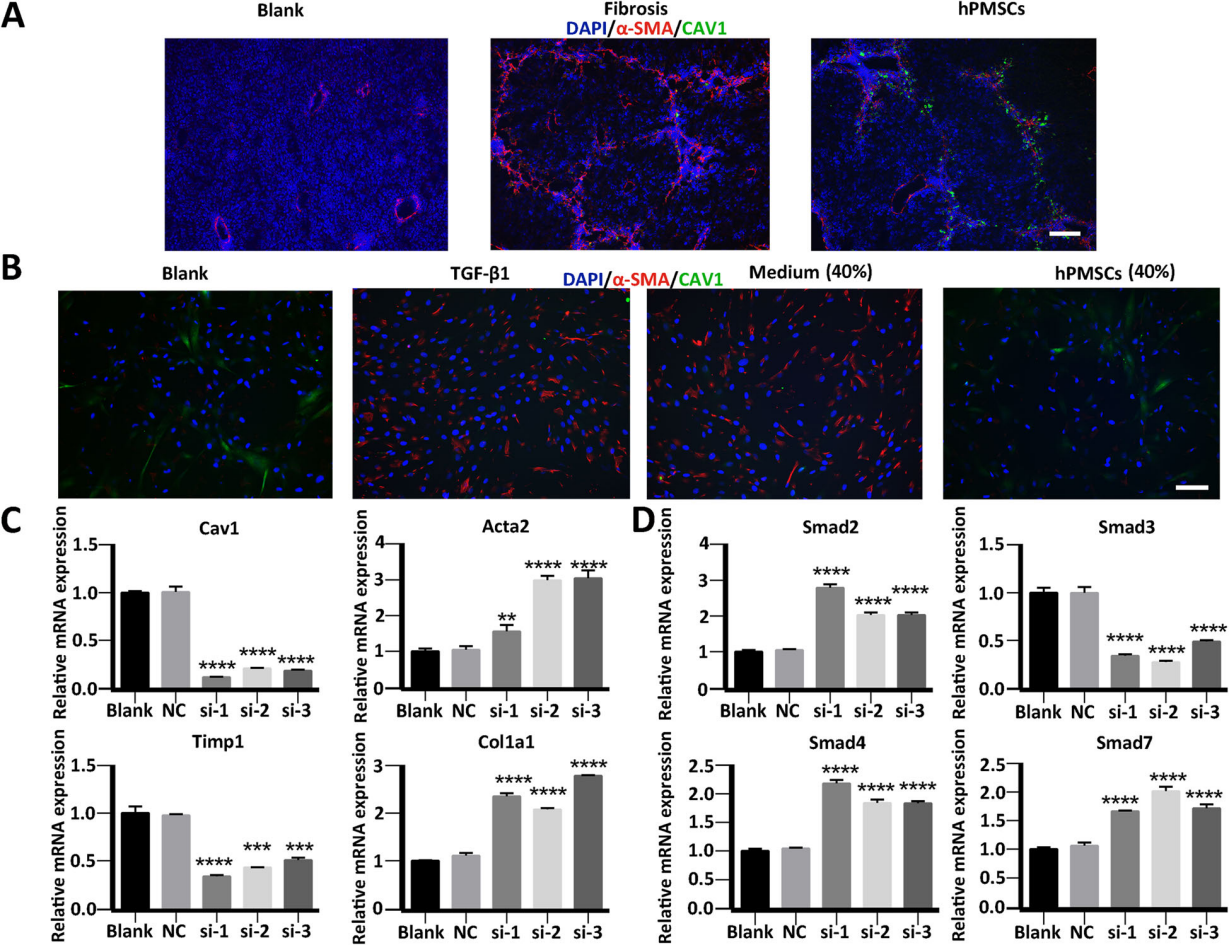
Upregulation of Cav1 after hPMSCs treatment was important in relieving liver fibrosis and inhibition of activated HSCs. **a** Immunohistochemistry staining using anti CAV1 antibody in liver sections. **b** Immunofluorescence staining using anti CAV1 antibody in HSCs. **c**, **d** Expression of fibrosis-related genes (**c**) and TGF-/Smad signaling pathway related genes (**d**) at different groups was determined using qRT-PCR. Relative mRNA expression was normalized to *-actin* and compared with the NC group. Cells from blank group were unactivated HSCs, cells from si (1-3) group or NC group were HSCs that transfected with *Cav1* siRNA (1-3) or *Cav1* siRNA-negative control. Scale bar, 50 m. ****p < 0.0001, ***p < 0.001, **p < 0.01, *p < 0.05; ns, no significance

To test this hypothesis, we investigated the effect of Cav1 on HSC activation. We measured the expression of Cav1 and -SMA in liver tissues by immunofluorescence staining. Results showed that Cav1 was expressed at a low level both in normal liver tissues and in fibrotic liver tissues, where -SMA was greatly upregulated after CCl_4_ administration. However, after treatment with hPMSCs, the expression of Cav1 was upregulated compared with that in fibrotic liver tissues, accompanied by the reduction of -SMA in the hepatic lobular margin (Fig. 6a). To further illustrate the relationship between Cav1 downregulation and HSC activation, we also tested the expression of Cav1 and -SMA in activated HSCs. Compared to unactivated HSCs, -SMA was significantly increased while Cav1 was decreased in activated HSCs. After treatment with hPMSC secretomes, -SMA levels were greatly attenuated, while Cav1 levels were partially restored in activated HSCs (Fig. 6b). These data demonstrated the involvement of Cav1 in HSC activation.

We then carried out loss-of-function experiments by transfecting an siRNA targeting human *Cav1*, which effectively reduced *Cav1* expression in HSCs. *Cav1* was not knocked down in siRNA-negative control (siRNA-NC)-transfected HSCs. Untransfected HSCs served as control cells (blank). HSC mRNAs from different groups were collected and subjected to qRT-PCR testing. Compared to control cells, the expression of pro-fibrotic genes, such as *Acta2*, and *Col1a1* were upregulated by 1.53-fold in *Cav1*-silenced HSCs, accompanied by the attenuation of *Timp1*, an anti-fibrotic gene (Fig. 6c), indicating a vital role of *Cav1* in HSC activation and collagen production. To explore the molecular mechanism of the anti-fibrotic effects of *Cav1*, we detected the regulation of Smad activation by *Cav1* in HSCs using loss-of-function experiments. It is noteworthy that Smad genes, including *Smad2* and *Smad4*, which are related to the TGF- signaling pathway, were upregulated in *Cav1*-silenced HSCs, but not in negative control group and control cells (Fig. 6d). These data reveal the involvement of the TGF-/Smad signaling pathway in Cav1-mediated HSC activation.

### hPMSCs inhibit HSC activation by restoring Cav1 function

**Fig. 7:**
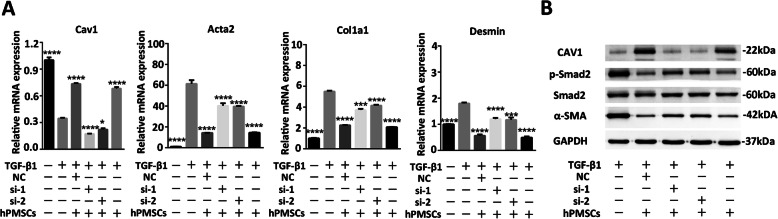
hPMSCs inhibit TGF-/Smad signaling pathway by restoring the function of Cav1 in HSCs. **a** HSCs were transfected with *Cav1* siRNA 1-2 and *Cav1* siRNA-NC, respectively. Transfected HSCs were treated with TGF-1 and then cultured with or without hPMSC secretomes (40%). Expression of *Cav1* and fibrosis-related genes (*Acta2, Col1a1, Desmin*) at different groups was determined using qRT-PCR. Relative mRNA expression was normalized to *-actin* and compared with the TGF-1 group (column 2). **b** Representative western blot of CAV1, -SMA, and Smad2 from HSCs at different groups. Cells from TGF-1 group were activated HSCs, without extra treatment. ****p < 0.0001, ***p < 0.001, **p < 0.01, *p < 0.05; ns, no significance

To explore the interplay between hPMSC and Cav1 in HSC activation, we prepared siRNA-transfected HSCs, cells were activated by TGF-1 and then treated with hPMSC secretomes (columns 36). Untransfected but activated HSCs served as control (column 2). Additionally, unactivated HSCs were also tested (column 1). The detailed operation is shown in Table S5. As shown in Fig. 7a, the expression of *Cav1* was reduced in HSCs in the presence of TGF-1. It is noteworthy that the decreased *Cav1* in activated HSCs can be upregulated by hPMSC secretomes. Knockdown of *Cav1* in activated HSCs, however, alleviated the effect of hPMSC secretomes on the upregulation of *Cav1* (Fig. 7a). Furthermore, the relative expression of the pro-fibrotic genes *Acta2*, *Col1a1*, and *Desmin* were also measured and normalized to *-actin*. The data showed that the trend of changes in pro-fibrotic gene expression in different groups was opposite to that of *Cav1* (Fig. 7a). These results indicate that hPMSC secretomes inhibit HSC activation by restoring *Cav1* function in activated HSCs.

We then collected protein from HSCs of different groups and validated the results by western blot analysis. Consistent with the results from the qRT-PCR assay, Cav1 was reduced in activated HSCs that were induced by TGF-1 (Fig. 7b). In contrast, hPMSC treatment upregulated Cav1 expression in activated HSCs and inhibited HSC activation, as indicated by the reduction of -SMA production. However, *Cav1* knockdown in activated HSCs attenuated the inhibitory effect of hPMSCs in activated HSCs (Fig. 7b). Importantly, after hPMSC treatment, HSCs showed reduced TGF-/Smad signaling, as reflected by a significantly smaller amount of phosphorylation of Smad2 and -SMA expression compared to that in the activated HSCs. However, knockdown of *Cav1* by siRNA increased Smad-2 phosphorylation and -SMA expression in activated HSCs even after treatment with hPMSC secretomes (Fig. 7b). In summary, hPMSC treatment restored the function of Cav1, and elevated Cav1 was sufficient to inhibit HSC activation and collagen production, partly by regulating the TGF-/Smad signaling pathway.

## Discussion

In this study, we demonstrated that transplantation of hPMSCs can reduce LF in a mouse model, with changes including the improvement of liver function, inhibition of inflammation, and a reduction in ECM deposition. Moreover, the therapeutic effects of hPMSCs against mild-to-moderate LF were significantly greater than those in severe fibrotic cases. Furthermore, our in vitro and in vivo data indicated that the therapeutic effects of hPMSCs are achieved partly through inhibition of the TGF-/Smad signaling pathway via upregulation of *Cav1* in activated HSCs, which resulted in inhibited HSC activation and alleviated LF.

In recent years, hPMSC-based cell therapy in regenerative research has gained a broad interest owing to its great potential for self-renewal, differentiation, and the immunomodulatory properties [19, 20]. Compared with the well-investigated autologous MSCs, including bone marrow and adipose mesenchymal stem cells, the number of studies using hPMSCs in the treatment of liver disease is relatively small, and the underlying molecular mechanisms have not yet been fully elucidated. In this study, we used mouse models of CCl_4_-injured LF to explore the therapeutic value of hPMSCs. Our findings indicated that hPMSC transplantation not only enhanced general hepatic function, as indicated by increased levels of liver functional index, including ALT, AST, and ALB, but also alleviated LF, as demonstrated by reductions in collagen fiber regions in liver tissues. This effect occurred concomitantly with a reduction in activated HSCs and downregulation of fibrosis-related genes. This is consistent with previous studies in a miniature pigs and rat models of CCl_4_-injured liver [21, 22]. Therefore, our findings provide further proof of the potential therapeutic effects of hPMSCs in LF.

Choosing an appropriate time window is a key factor for hPMSC transplantation. Although the effects of hPMSCs in LF have been reported in previous studies [31], to date, little is known about the optimal of therapeutic procedures in terms of time windows. In previous studies, MSCs were transplanted at 4 weeks after CCl_4_ administration [26]. However, in a few studies, MSCs were administered at 6 weeks after CCl_4_ administration, at the more severe stage of the LF according to liver function tests and histopathological examination [25]. In this study, two representative time points were chosen and comparative research was performed. We found that the therapeutic effects of hPMSCs at the early stage of LF were significantly greater than those of hPMSCs in advanced LF. These results suggest that earlier intervention with hPMSCs, may afford better therapeutic effects. These findings will need to be considered in the design of future clinical studies. In addition, to explore the effectiveness of different infusion doses of hPMSCs to LF, we adopted two commonly used doses (high and low), based on other trials that reported improved LF [12]. Our results showed that both doses improved LF and restored all indicators, with no significant difference between them. This provided a reference for our selection of the minimum effective dose (MED) for MSC treatment, for use in clinical trials [32].

In the present study, we also investigated the possible mechanisms involved in relieving LF by hPMSCs. In summary, the following mechanisms may account for them: Transplanted hPMSCs inhibit the secretion of multiple cytokines that otherwise promote inflammation and impair liver restoration [6, 15, 33]. Serum IL-6 and TNF- levels were significantly lower in hPMSC-treated mice than in untreated fibrotic mice. Furthermore, the number of Kupffer cells in the fibrotic liver also decreased after hPMSC treatment. These results provide evidence supporting the immunomodulatory roles of hPMSCs, which is beneficial to the improvement of liver function as well as the inhibition of LF. In addition, the therapeutic potential of hPMSCs in LF stems mainly from inhibiting HSC activation. According to RNA-seq analysis of liver tissues and GO enrichment analysis (Figure S5), we found that hPMSC treatment contributed to the upregulation of *Cav1* in activated HSCs, which then helped in the inhibition of HSC activation by regulating the TGF-1/Smad signaling pathway.

CAV1 is a fatty acid- and cholesterol-binding protein that constitutes the major structural protein of caveolae [30, 34]. Previous studies have confirmed that CAV1 can exercise a homeostatic function in the process of fibrosis by regulating TGF- and its downstream signaling [35, 36]. Moreover, *Cav1* knockout mice have revealed impaired wound healing and profound fibrosis in the lungs, heart, and liver [36,38]. However, it remains unclear whether *Cav1*-mediated signaling pathways play an important role in relieving LF by hPMSC treatment. In this study, we found that mRNA and protein levels of *Cav1* were decreased in activated HSCs. Importantly, *Cav1* upregulation can be achieved after hPMSC treatment. *Cav1* influenced the activity of Smad2, Smad3, and Smad4. In particular, it influenced the phosphorylation of Smad2, which then inhibits the TGF-1/Smad signaling pathway as well as HSC activation. While endogenous inhibition of *Cav1* by siRNA alleviated the effect of hPMSC secretomes on the upregulation of *Cav1*, based on immunofluorescence staining and qRT-PCR assays. The results from in vitro assays were consistent with the findings in our animal models.

In summary, these combined mechanisms contribute significantly to the therapeutic potential of hPMSCs in the treatment of LF. To our knowledge, this is the first report to support the important role of *Cav1* in MSC-based therapy for LF. Two limitations were also present in this study. First, we did not verify our findings in clinical samples because LF tissue is scarce. Second, it remains unclear whether anti-fibrotic factors or hPMSC-derived exosomes contribute to *Cav1* upregulation after hPMSC treatment and the underlying mechanisms. These points will be considered in further studies.

## Conclusions

Collectively, we present further evidence demonstrating the potential of hPMSCs in treating LF. The injection of hPMSCs at conventional doses at the mild-to-moderate stage of experimental LF had a significant therapeutic effect. Moreover, hPMSC-mediated upregulation of *Cav1* in activated HSCs plays a key role in deactivating HSCs via inhibition of TGF-1/Smad signaling. These findings will contribute to the development of effective treatment for fibrotic liver diseases.

## Supplementary Information

**Additional file 1: Figure S1.** Isolation and characterization of human placental mesenchymal stem cells (hPMSCs). **a** Image of primary hPMSCs, passage (P)5 hPMSCs, and passage 10 hPMSCs. The cells showed homogenous fibroblastic morphology. **b** Expression of cell surface markers on hPMSCs. hPMSCs possessed the surface marker profile typical for mesenchymal stem cells, and were positive for the mesenchymal markers CD73, CD90, CD166 and CD105, and negative for the hematopoietic and endothelial markers CD45, CD34, CD11b, and showed almost no expression of HLA-DR. **c** Multiple differentiation potential of hPMSCs. Under specific induction conditions, they could differentiate into adipose cells, osteocytes and chondrocytes. Scale bar: 50m.

**Additional file 2: Figure S2.** The development of CCl_4_-injured liver fibrosis in mice. **a** Diagram of establishing hepatic fibrosis model in mice. CCl_4_ was administered twice a week for 6 weeks. **b** liver sections stained with Sirius red (upper) and Masson trichome (bottom) on week 0, 2, 4, 6 respectively. **c** Immunofluorescence staining using anti -SMA (red) antibodies and DAPI (blue) on week 0, 2, 4, 6 respectively. **d** Hepatic function was assessed by serum level of AST, ALT, ALB and hepatic hydroxyproline content in liver tissues were measured in CCl_4_-injured mice on week 0, 2, 4, 6. Scale bar: 50m. ****, *p* < 0.0001; ***, *p* < 0.001; **, *p* < 0.01; *, *p* < 0.05; ns, no significance.

**Additional file 3: Figure S3.** Experimental conditions for the activation of HSCs by TGF-1. Representative western blot of -SMA and GAPDH from HSCs at different groups. The four samples of HSCs on the left side (column 1-4) were from starved treatment group, cells were pretreated with the starvation medium (DMEM containing 0.2% FBS) for 24 h and then cultured in human stellate cell medium containing different concentrations of TGF-1. The four samples of HSCs on the right side (column 5-8) were from the non-starved treatment group, these cells cultured in human stellate cell medium containing different concentrations of TGF-1.

**Additional file 4: Figure S4.** Co-culture with MSC significantly reduces the activation of HSC. **a** Representative western blot of -SMA and GAPDH on activated HSCs under co-culture conditions with hPMSCs. **b** Typical cell morphology (upper) and -SMA immunofluorescence staining (lower) of HSCs. **c** Expression of fibrosis-related genes in activated HSCs was determined using qRT-PCR. Relative mRNA expression was normalized to *-actin*, and compared with the TGF-1 group. Cells from blank group were un-activated HSCs, cells from TGF-1 group were activated HSCs that induced by TGF-1, cells from hPMSCs group were co-cultured with hPMSCs. Scale bar: 50m. ****, *p* < 0.0001; ***, *p* < 0.001; **, *p* < 0.01; *, *p* < 0.05; ns, no significance. HSCs, hepatic stellate cells.

**Additional file 5: Figure S5.** Screening and verification of potential functional genes. A. Expression of potential functional genes of liver tissues. B. Expression of potential functional genes of HSCs. C. Verification of Caveolin-1 gene expression in different treatment HSCs in vitro. Data are shown as means SEM. Statistical significance was assessed by unpaired, two-tailed Students t test. ***, *p* < 0.001;**, *p* < 0.01; *, *p* < 0.05; ns, no significance.

**Additional file 6: Table S1.** antibodies used for immunofluorescence, FACS analysis and WB. **Table S2.** Quantitative RT-PCR primer sequences of mouse. **Table S3.** Quantitative RT-PCR primer sequences of human. **Table S4.** Small interfering RNA of Caveloin1. **Table S5.** Effects of secretomes of hPMSCs on HSCs activation and silencing Caveolin1 expression simultaneously.

## Data Availability

The datasets used and/or analyzed during the current study are available from the corresponding author on reasonable request

## Abbreviations

MSCs: Mesenchymal stem cells
hPMSCs: Human placental mesenchymal stem cells
TGF-1: Transforming growth factor 1
PBS: Phosphate-buffered saline
FBS: Fetal bovine serum
ECM: Extracellular matrix
HSCs: Hepatic stellate cells
ELISA: Enzyme-linked immunosorbent assay
PCR: Polymerase chain reaction
RT-PCR: Reverse transcription-polymerase chain reaction
qRT-PCR: Quantitative-reverse transcription-PCR
GAPDH: Glyceraldehyde-3-phosphate dehydrogenase
CCl_4_: Carbon tetrachloride
-SMA: Alpha smooth muscle Actin
AST: Aspartate aminotransferase
ALT: Alanine aminotransferase
ALB: Albumin
IL-6: Interleukin-6
IFN-: Interferon-
TNF-: Tumor necrosis factor-
Acta2: Actin alpha 2
Timp1: Tissue Inhibitor Of Metalloproteinases 1
Col1a1: Collagen Type I Alpha 1
siRNA: Small interfering RNA
CAV1: Caveolin1
GO: Gene Ontology
WB: Western blot
KC: Kupffer cells
MoMF: Monocyte-derived macrophage
TM: Treatment with hPMSCs in the mild-to-moderate stage of LF
TS: Treatment with hPMSCs in the severe stage of LF
TM_high_: Treatment with a high dose of hPMSCs in the mild-to-moderate stage of LF
TM_low_: Treatment with a low dose of hPMSCs in the mild-to-moderate stage of LF
TS_high_: Treatment with a high dose of hPMSCs in the severe stage of LF
TS_low_: Treatment with a low dose of hPMSCs in the severe stage of LF
