# Supplementary figures and images for: Human placental mesenchymal stem cells ameliorate liver fibrosis in mice by upregulation of Caveolin1 in hepatic stellate cells
Source: Stem Cell Res Ther. 2021 May 20;12:294. doi: 10.1186/s13287-021-02358-x (PMC8139101; doi:10.1186/s13287-021-02358-x)

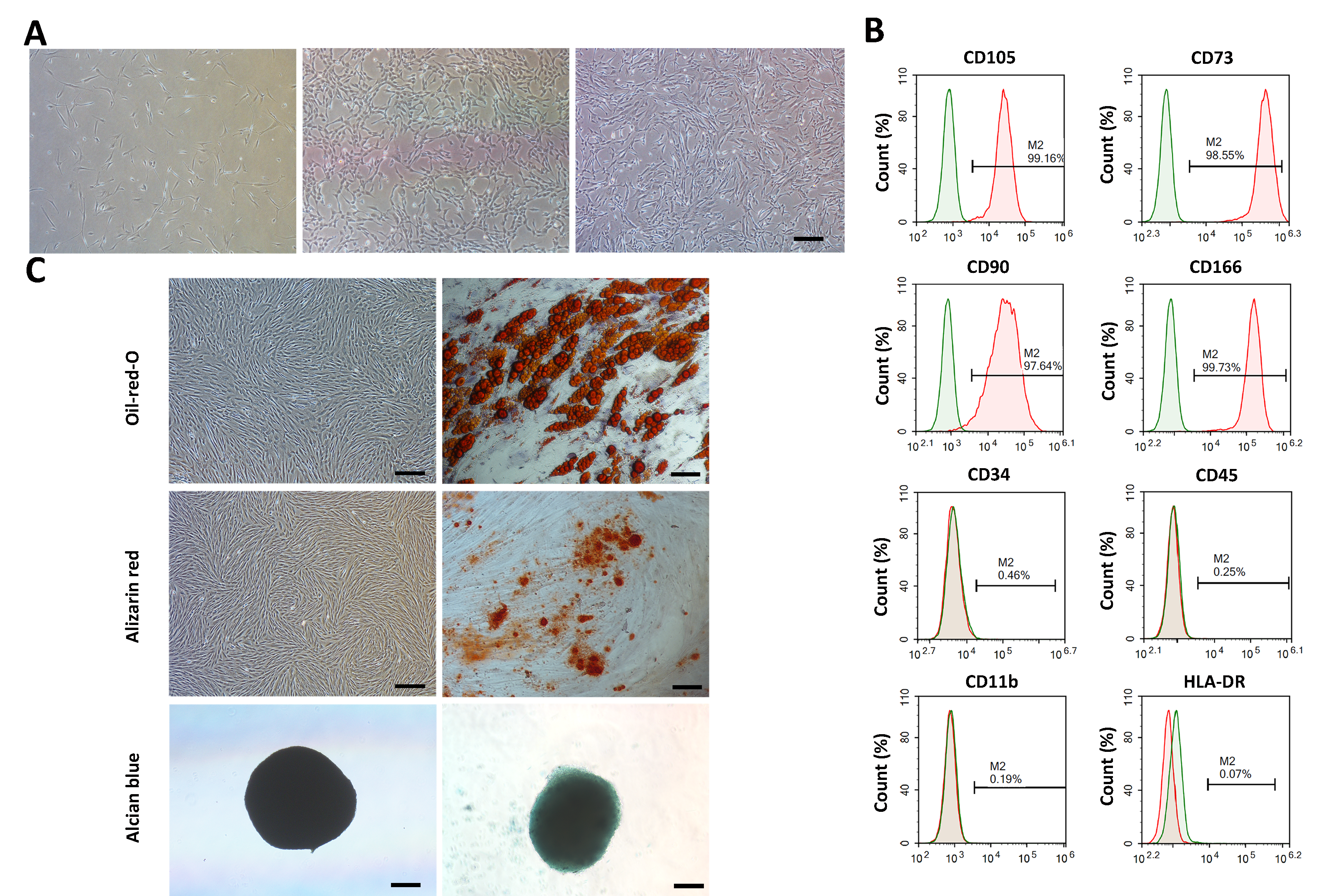

Supplement: Supplementary file 1 — Additional file 1: Figure S1. Isolation and characterization of human placental mesenchymal stem cells (hPMSCs). a Image of primary hPMSCs, passage (P)5 hPMSCs, and passage 10 hPMSCs. The cells showed homogenous fibroblastic morphology. b Expression of cell surface markers on hPMSCs. hPMSCs possessed the surface marker profile typical for mesenchymal stem cells, and were positive for the mesenchymal markers CD73, CD90, CD166 and CD105, and negative for the hematopoietic and endothelial markers CD45, CD34, CD11b, and showed almost no expression of HLA-DR. c Multiple differentiation potential of hPMSCs. Under specific induction conditions, they could differentiate into adipose cells, osteocytes and chondrocytes. Scale bar: 50m. [file 13287_2021_2358_MOESM1_ESM.tif]

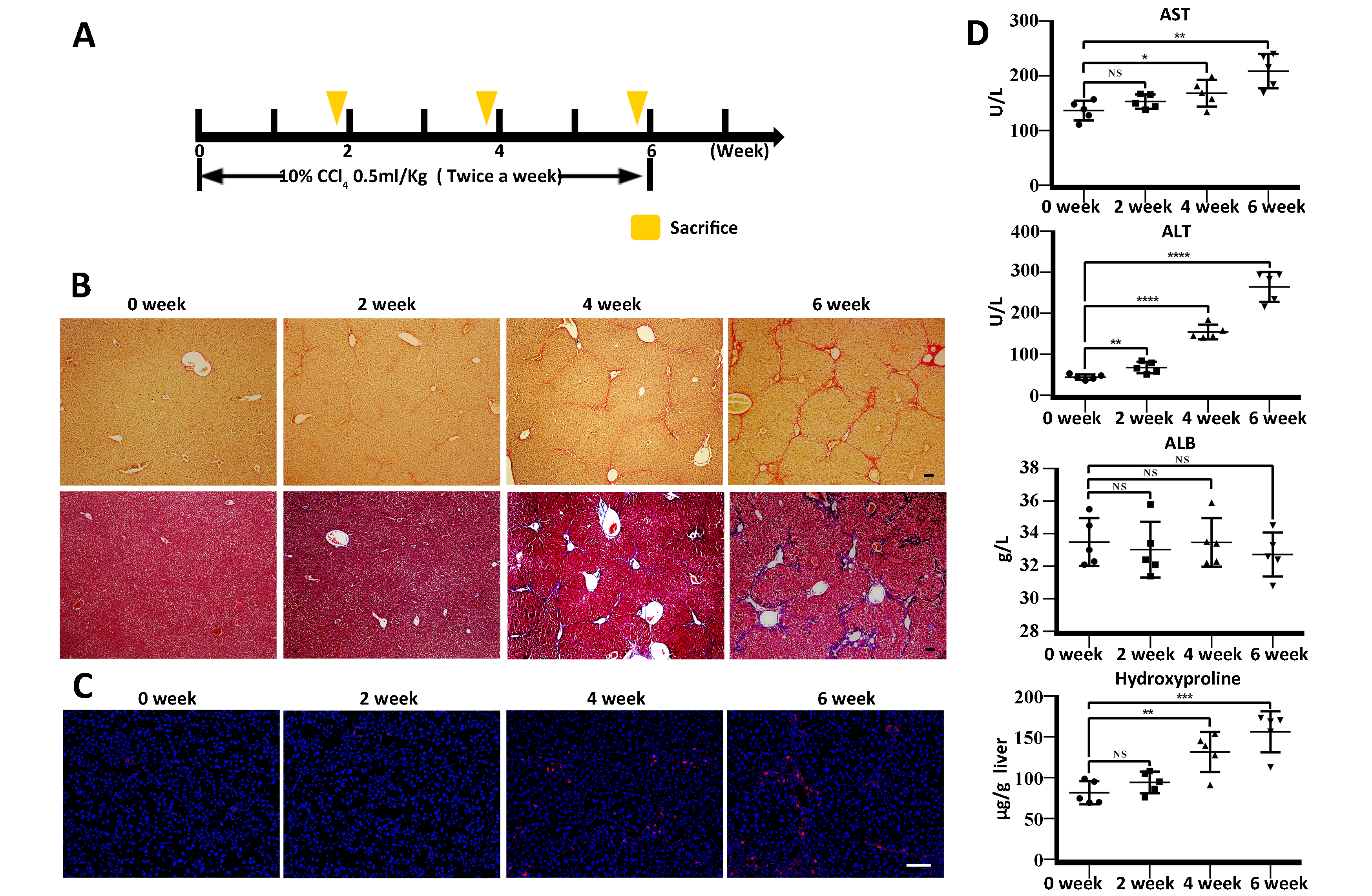

Supplement: Supplementary file 2 — Additional file 2: Figure S2. The development of CCl4-injured liver fibrosis in mice. a Diagram of establishing hepatic fibrosis model in mice. CCl4 was administered twice a week for 6 weeks. b liver sections stained with Sirius red (upper) and Masson trichome (bottom) on week 0, 2, 4, 6 respectively. c Immunofluorescence staining using anti -SMA (red) antibodies and DAPI (blue) on week 0, 2, 4, 6 respectively. d Hepatic function was assessed by serum level of AST, ALT, ALB and hepatic hydroxyproline content in liver tissues were measured in CCl4-injured mice on week 0, 2, 4, 6. Scale bar: 50m. ****, p < 0.0001; ***, p < 0.001; **, p < 0.01; *, p < 0.05; ns, no significance. [file 13287_2021_2358_MOESM2_ESM.tif]

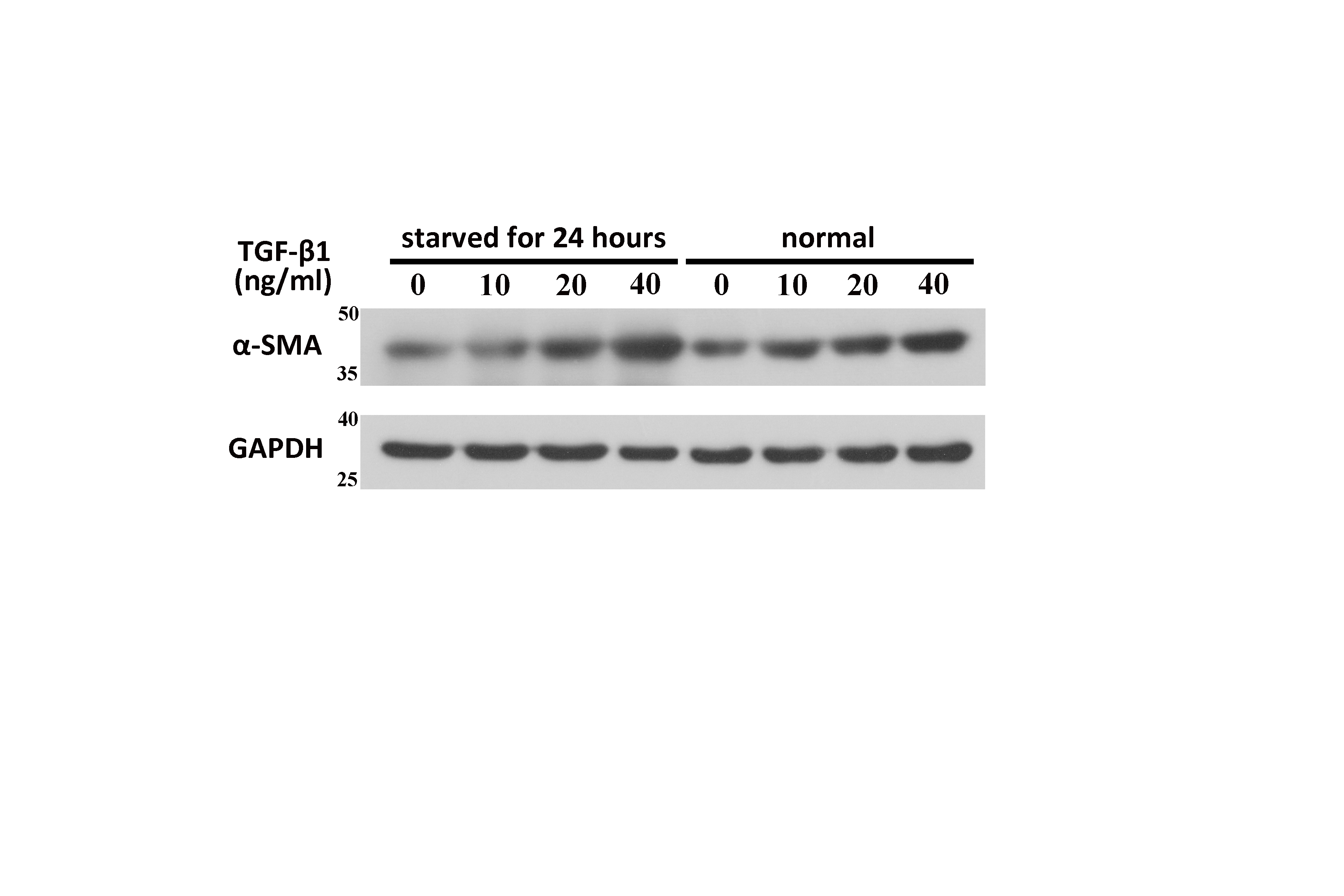

Supplement: Supplementary file 3 — Additional file 3: Figure S3. Experimental conditions for the activation of HSCs by TGF-1. Representative western blot of -SMA and GAPDH from HSCs at different groups. The four samples of HSCs on the left side (column 1-4) were from starved treatment group, cells were pretreated with the starvation medium (DMEM containing 0.2% FBS) for 24 h and then cultured in human stellate cell medium containing different concentrations of TGF-1. The four samples of HSCs on the right side (column 5-8) were from the non-starved treatment group, these cells cultured in human stellate cell medium containing different concentrations of TGF-1. [file 13287_2021_2358_MOESM3_ESM.tif]

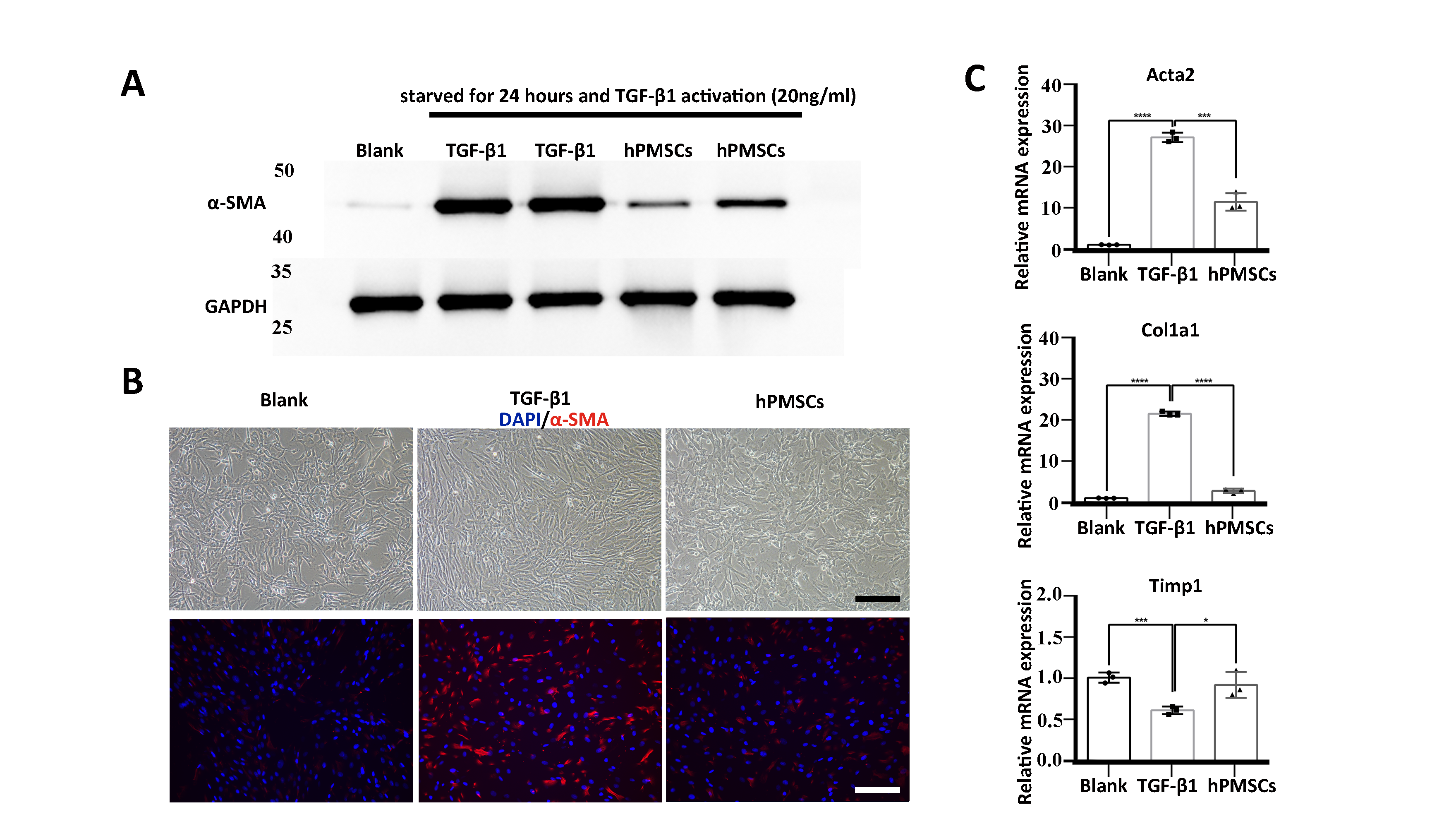

Supplement: Supplementary file 4 — Additional file 4: Figure S4. Co-culture with MSC significantly reduces the activation of HSC. a Representative western blot of -SMA and GAPDH on activated HSCs under co-culture conditions with hPMSCs. b Typical cell morphology (upper) and -SMA immunofluorescence staining (lower) of HSCs. c Expression of fibrosis-related genes in activated HSCs was determined using qRT-PCR. Relative mRNA expression was normalized to -actin, and compared with the TGF-1 group. Cells from blank group were un-activated HSCs, cells from TGF-1 group were activated HSCs that induced by TGF-1, cells from hPMSCs group were co-cultured with hPMSCs. Scale bar: 50m. ****, p < 0.0001; ***, p < 0.001; **, p < 0.01; *, p < 0.05; ns, no significance. HSCs, hepatic stellate cells. [file 13287_2021_2358_MOESM4_ESM.tif]

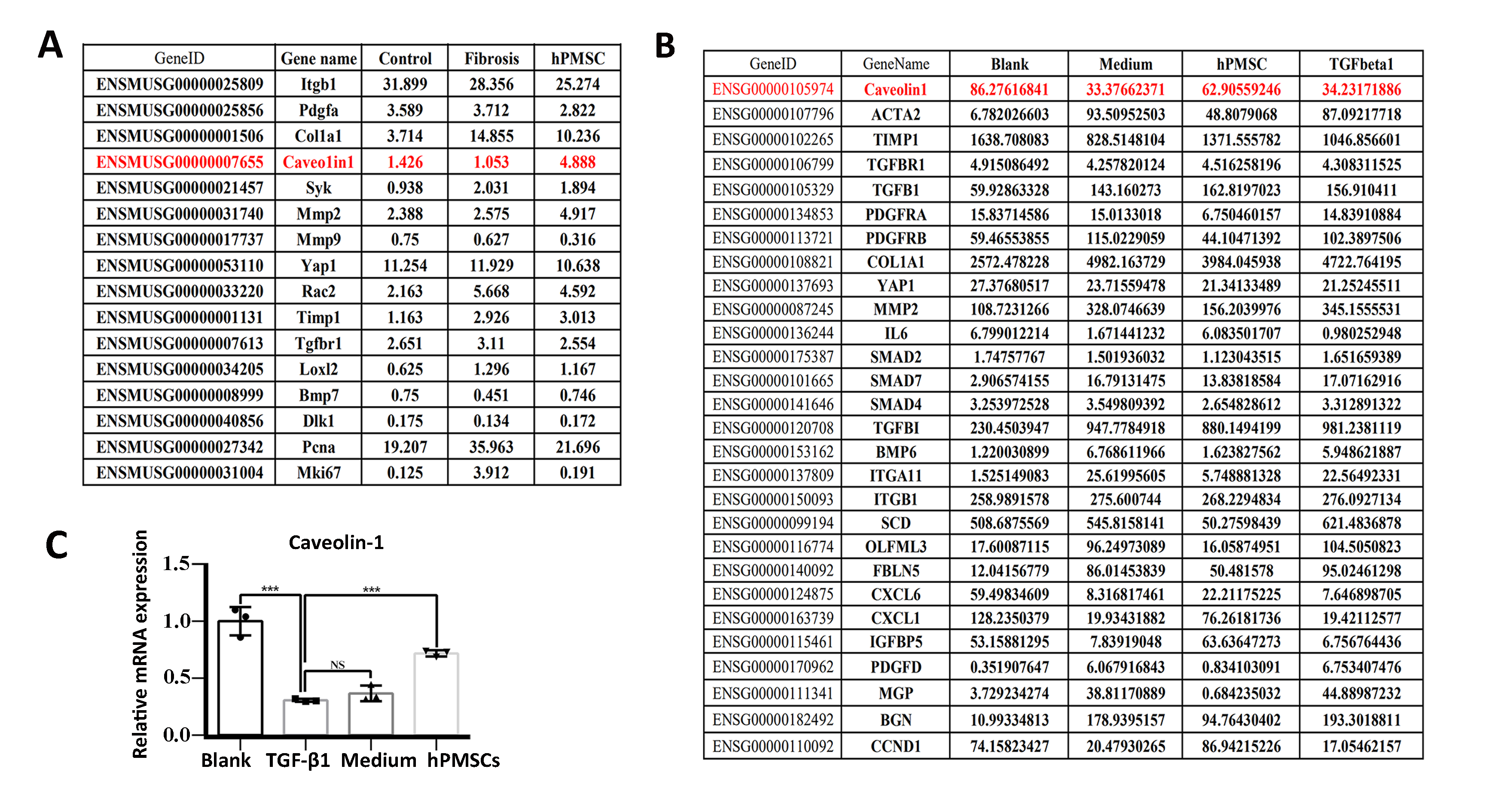

Supplement: Supplementary file 5 — Additional file 5: Figure S5. Screening and verification of potential functional genes. A. Expression of potential functional genes of liver tissues. B. Expression of potential functional genes of HSCs. C. Verification of Caveolin-1 gene expression in different treatment HSCs in vitro. Data are shown as means SEM. Statistical significance was assessed by unpaired, two-tailed Students t test. ***, p < 0.001;**, p < 0.01; *, p < 0.05; ns, no significance. [file 13287_2021_2358_MOESM5_ESM.tif]
