# Supplementary material for: Human placental mesenchymal stem cells ameliorate liver fibrosis in mice by upregulation of Caveolin1 in hepatic stellate cells
Source: Stem Cell Res Ther. 2021 May 20;12:294. doi: 10.1186/s13287-021-02358-x (PMC8139101; doi:10.1186/s13287-021-02358-x)
Supplement: Supplementary file 6 — Additional file 6: Table S1. antibodies used for immunofluorescence, FACS analysis and WB. Table S2. Quantitative RT-PCR primer sequences of mouse. Table S3. Quantitative RT-PCR primer sequences of human. Table S4. Small interfering RNA of Caveloin1. Table S5. Effects of secretomes of hPMSCs on HSCs activation and silencing Caveolin1 expression simultaneously. [file 13287_2021_2358_MOESM6_ESM.docx]

**Table S1. antibodies used for immunofluorescence, FACS analysis and WB**

| Anti-alpha smooth muscle Actin mAb | Abcam |
| --- | --- |
| Anti-Caveolin-1 mAb | Abcam |
| Phospho-SMAD2 (Ser465/Ser467) Rabbit mAb | Cell Signaling Technology |
| Smad2 (D43B4) XP^®^ Rabbit mAb | Cell Signaling Technology |
| mouse anti-human GAPDH | Santa Cruz Biotechnology |
| FITC-anti-human CD105 | Biolegend |
| FITC-Mouse IgG1,κ | Biolegend |
| PE-anti-human HLA-DR | Biolegend |
| PE-Mouse IgG2a, κ | Biolegend |
| PE- anti-human CD34 | Biolegend |
| PE- Mouse IgG1,κ | Biolegend |
| PE- anti-human CD166 | Biolegend |
| PE- Mouse IgG1,κ | Biolegend |
| PE- anti-human CD45 | Biolegend |
| PE-Mouse IgG2b, κ | Biolegend |
| FITC-anti-human CD90 | Biolegend |
| APC- anti-mouse CD45 | Biolegend |
| PE/Cy7- anti-mouse CD11b | Biolegend |
| FITC- anti-mouse F4/80 | Biolegend |

**Table S2. Quantitative RT-PCR primer sequences of mouse**

| Gene name | Sequence (5’ to 3’) |
| --- | --- |
| Acta2-F | GTACCACCATGTACCCAGGC |
| Acta2-R | GCTGGAAGGTAGACAGCGAA |
| Timp1-F | CCAGAACCGCAGTGAAGAGT |
| Timp1-R | GTACGCCAGGGAACCAAGAA |
| Desmin-F | AGTTCAATGACATCCCGCGT |
| Desmin-R | AACTCCTGGTTCACAGCGTC |
| Col1a1-F | GAGAGGTGAACAAGGTCCCG |
| Col1a1-R | AAACCTCTCTCGCCTCTTGC |
| Vimentin-F | TGGCACGTCTTGACCTTGAA |
| Vimentin-R | CACGCTTTCATACTGCTGGC |
| β-Actin-F | ATATCGCTGCGCTGGTCG |
| β-Actin-R | CGATGGAGGGGAATACAGCC |

**Table S3. Quantitative RT-PCR primer sequences of human**

| Gene name | Sequence (5’ to 3’) |
| --- | --- |
| Acta2-F | CCTGACTGAGCGTGGCTATT |
| Acta2-R | GCCCATCAGGCAACTCGTAA |
| Timp1-F | ACACTGTTGGCTGTGAGGAAT |
| Timp1-R | CCTTTTCAGAGCCTTGGAGGA |
| PDGF-F | ACTCCAGGTGTCATCCATCAAC |
| PDGF-R | TTCTTTGCGGGGGTATGTCC |
| Col1a1-F | CCCCGAGGCTCTGAAGGT |
| Col1a1-R | CACCAGCAATACCAGGAGCA |
| TGF-β1-F | TCAGCTCCACGGAGAAGAAC |
| TGF-β1-R | AGAAGTTGGCATGGTAGCCC |
| β-Actin-F | TGCCGACAGGATGCAGAAG |
| β-Actin-R | CACATCTGCTGGAAGGTGGA |
| Desmin-F | AACTTCCGAGAAACCAGCCC |
| Desmin-R | ATCCCGTGTCTCGATGGTCT |
| Vimentin-F | GGACCAGCTAACCAACGACA |
| Vimentin-R | AAGGTCAAGACGTGCCAGAG |
| GAPDH1-F | CAAATTCCATGGCACCGTCAA |
| GAPDH1-R | AGCATCGCCCCACTTGATTT |
| SMAD2-F | TGAAGAGACTGCTGGGATGG |
| SMAD2-R | TTCTTCCTGCCCATTCTGCT |
| SMAD3-F | ACCGGAAAGCATGGTGGATG |
| SMAD3-R | GTGGTAGGGATTCACGCAGA |
| SMAD4-F | TGTTGATGACCTTCGTCGCT |
| SMAD4-R | GGTCTGCAATCGGCATGGTA |
| SMAD6-F | AGTGACTGCGAGACGGTG |
| SMAD6-R | TACGTGACGGTTTTGAGTTCCT |
| SMAD7-F | TCCTCGGAAGTCAAGAGGCT |
| SMAD7-R | GGACAGTCTGCAGTTGGTTTG |
| CAV1-F | CAGAACCAGAAGGGACACACA |
| CAV1-R | GCAGACAGCAAGCGGTAAAA |

**Table S4. Small interfering RNA of Caveloin1**

Synthesized sheet of Oligo

| Serial number | Oligo’ name | Sequence（5’-3’） | | 5’label |
| --- | --- | --- | --- | --- |
| 1 | CAV1-siR  NA-1 | Sense strand | CCAGAAGGGACACACAGUUdTdT | N/A |
|  |  | Antisense strand | AACUGUGUGUCCCUUCUGGdTdT | N/A |
| 2 | CAV1-siR  NA-2 | Sense strand | UAGAUGGAAUAGACACGGCdTdT | N/A |
|  |  | Antisense strand | GCCGUGUCUAUUCCAUCUAdTdT | N/A |
| 3 | CAV1-siR  NA-3 | Sense strand | CCUUCACUGUGACGAAAUAdTdT | N/A |
|  |  | Antisense strand | UAUUUCGUCACAGUGAAGGdTdT | N/A |
| 4 | NC | Sense strand | ACGUGACACGUUCGGAGAAdTdT | N/A |
|  |  | Antisense strand | UUCUCCGAACGUGUCACGUdTdT | N/A |
| 6 | GAPDH | Sense strand | GUGGAGAUUGUUGCCAUCAdTdT | N/A |
|  |  | Antisense strand | UGAUGGCAACAAUCUCCACdTdT | N/A |

**Table S5. Effects of secretomes of hPMSCs on HSCs activation and silencing Caveolin1 expression simultaneously**

| Number  Reagent | 1 | 2 | 3 | 4 | 5 | 6 |
| --- | --- | --- | --- | --- | --- | --- |
| 2% FBS DMEM | — | 2.0ml | 1.35ml | 1.35ml | 1.35ml | 1.6ml |
| TGF-β1 | — | 40μl | 40μl | 40μl | 40μl | 40μl |
| siRNA-NC | — | — | 250μl | — | — | — |
| siRNA1 | — | — | — | 250μl | — | — |
| siRNA2 | — | — | — | — | 250μl | — |
| hPMSCs | — | — | 400μl | 400μl | 400μl | 400μl |
